# Evaluation of decision support to wean patients from mechanical ventilation in intensive care: a prospective study reporting clinical and physiological outcomes

**DOI:** 10.1007/s10877-024-01231-5

**Published:** 2024-11-09

**Authors:** Marcela P. Vizcaychipi, Dan S. Karbing, Laura Martins, Amandeep Gupta, Jeronimo Moreno-Cuesta, Manu Naik, Ingeborg Welters, Suveer Singh, Georgina Randell, Leyla Osman, Stephen E. Rees

**Affiliations:** 1 Magill Department of Anaesthesia and Intensive Care Medicine, Chelsea and Westminster Hospital, APMIC, Surgery & Cancer, Imperial College London, London, UK; 2 Department of Health Science and Technology, Aalborg University, Aalborg, Denmark; 3 Research and Development Delivery Team, Chelsea & Westminster Hospital, London, UK; 4 Intensive Care Unit, West Middlesex University Hospital, London, UK; 5 Intensive Care Unit, North Middlesex University Hospital, London, UK; 6 Norwich and Norfolk University Hospital Foundation Trust, Norwich, UK; 7 Intensive Care Unit, Liverpool University Hospital NHS Foundation Trust, Liverpool, UK; 8 Guys & St Thomas Hospital, London, UK

**Keywords:** Mechanical ventilation, Decision support, Randomised control trial

## Abstract

This study investigated the clinical and physiological response to use of the BEACON Caresystem, a bedside open-loop decision support system providing advice to guide clinicians when weaning patients from invasive mechanical ventilation. Multicenter prospective study conducted in five adult intensive care units in the UK. Following screening and assent, intubated patients mechanically ventilated for > 24 h were randomized to intervention or usual care. Intervention consisted of application of the BEACON Caresystem’s advice on tidal volume/inspiratory pressure, inspired oxygen, respiratory rate and PEEP. Usual care was defined as local clinical practice. The primary outcome was duration of mechanical ventilation. Secondary outcomes quantified prolonged intubation and survival; adverse events; ventilator settings and physiological state; time spent in ventilator modes; links to other therapy; the frequency of advice utilization and time spent outside normal physiological limits. The study was terminated early with a total of 112 patients included. Fifty-four were randomised to the intervention arm and fifty-eight to usual care. The study was underpowered and no significant differences were seen in duration of mechanical ventilation (p = 0.773), prolonged intubation or survival. Intervention arm patients had lower rates of adverse events (p = 0.016), including fewer hypoxaemic events (p = 0.008) and lower values of PEEP (p = 0.030) and tidal volume (p = 0.042). Values of peak inspiratory pressure and pressure support were reduced but at the boarder of statistical significance (p = 0.104, p = 0.093, respectively). No differences were seen for time in ventilator mode or other therapy. Advice presented by the decision support system was applied at the beside an average of 88% of occasions, with a significantly increased number of changes only in inspired oxygen fraction. No significant differences were seen in time spent outside physiological limits. This study investigated the use of the BEACON Caresystem, an open loop clinical decision support system providing advice on ventilator settings. It was terminated early, with no significant difference shown in duration of mechanical ventilation, the primary outcome. Application of advice indicated potential for fewer adverse events and improved physiological status. (*Trial registration* ClinicalTrials.gov under NCT03249623. Registered 22nd June 2017)

**Supplementary Information:**

The online version contains supplementary material available at 10.1007/s10877-024-01231-5.

## Introduction

Weaning from mechanical ventilation is a complex process requiring provision of adequate support, while ensuring rapid extubation and prevention of ventilator-associated complications [1, 2]. Guidelines are available [1] but are not always systematically applied [3], highlighting a need for decision support.

Computer systems can aid in selecting the appropriate settings for mechanical ventilation by application of protocols as rules [4–7], protocols/rules combined with artificial neural networks [8, 9] or physiological-model based approaches [10–12]. These systems can be either closed-loop, directly controlling the ventilator [6, 7, 10, 11], or open-loop where advice is provided to the attending physician [4, 5, 8, 9, 12].

The BEACON Caresystem (BC) is a physiological model-based decision support system that provides open-loop advice on mechanical ventilator settings. Advice is based on an extensive set of measurements, including volumetric capnography and indirect calorimetry, along with physiological mathematical models [12] including pulmonary mechanics and gas exchange; acid–base; respiratory drive; metabolism and circulation (see electronic supplementary material (ESM), S.2). Application of the BC is intended to provide a complete physiological model-based description of the individual patient which, when combined with decision theoretic models of clinical preference [13], might aid in the weaning of patients. The BC has previously been evaluated over short durations of time from 4 to 8 h [14, 15]. Over this duration, it has been shown to significantly reduce ventilation to levels which might be considered safe and beneficial [14] and to act to preserve respiratory muscle function [15].

This multi-centre study is a prospective, randomized, control trial to evaluate the use of the BC over the complete duration of invasive mechanical ventilation, in patients admitted to general adult intensive care units and having received mechanical ventilation for more than 24 h. The study was designed to evaluate whether advice provided by the system changed the duration of mechanical ventilation, with secondary outcomes quantifying prolonged intubation and survival; adverse events; ventilator settings and physiological state; time spent in ventilator modes; links to other therapy; the frequency of advice utilization and time spent outside normal physiological limits [16].

## Methods and analysis

The study hypothesis was that application of advice from the BC would reduce the duration of mechanical ventilation to successful extubation. This was quantified as the duration of mechanical ventilation, either from intubation as primary outcome, or from randomization as a secondary outcome. In addition, to understand whether re-intubation impacted ventilation duration, duration from randomization to first extubation was calculated. Secondary outcomes investigated prolonged intubation and survival, the number of adverse events and the ventilator settings applied and resulting physiological state, both for mandatory or supported ventilator modes. In addition, secondary outcomes quantified the time spent in mandatory or support modes, the initiation and success of spontaneous breathing tests (SBT), and whether the use of sedation, inotropes of neuromuscular blocking agents was different between arms. Application of the BC advice was optional allowing assessment of advice uptake. To understand the utilization of advice in the intervention arm, advice provided by the system was compared to changes in ventilator settings for a 2 h window following advice. Three primary categories were defined as to the user’s response to advice: followed; not acted on; or settings different than advised. As an additional assessment of advice use, the number of ventilator changes were counted and compared between groups. Full details are provided in the supplementary material (ESM S.10). A post-hoc analysis was also performed to asses differences in the percentage of time where physiological variables were above or below thresholds values considered to be detrimental. A full list of outcomes is provided in [17] and the ESM (ESM S.3). All outcomes reported in the study design are analysed and reported here, but with care taken in interpretation due to the underpowered nature of the study.

Extensive user training was provided, including standard operating procedures, education of super-users, training and refresher sessions and assessment of user competence. A 24/7 hotline was available for support. All training and support was provided by experts in the system from either the manufacturer or research team. Further details are provided in the supplementary material (ESM S.7).

All patients were screened for eligibility on admission to the ICU by the research team. Following screening, consent was obtained either from the patient where capable, a relative/personal consultee, or if not possible, a nominated professional consultee from outside the research group. Patients were randomized into the intervention arm, where BC advice was available, or to usual care (see ESM, Fig. S.1.1). Patients were included if adult, had > 24 h invasive mechanical ventilation; if the ventilator and mode was supported by the BC; and if haemodynamically stable. Patients were excluded according to the criteria in the ESM (ESM S.4), which includes: history of home ventilation or multiple ICU admissions; if catagorised as patient with a primary neurological disorder or with head trauma; on the presence of severe heart failure, end stage liver disease or morbid obesity; or if clinical conditions required treatment with ECMO. Patients randomized to either arm were connected to the BC system via a flow and gas sensor connection placed in the main ventilation flow to and from the patient (see Measurements).

The core of the BC is a set of physiological models describing pulmonary gas exchange, acid–base chemistry, lung mechanics, respiratory drive, metabolism and circulation. The system continuously monitors the patient’s ventilatory flow and hence volume, respiratory pressures, inspiratory and expiratory oxygen and carbon dioxide levels, and pulse oximetry measures of oxygen saturation. Periodic measurements of arterial blood gases are manually entered into the system. These measurements are applied by the BC to tune the mathematical physiological models to the individual patient, such that the models accurately describe current measurements. Once tuned, the models are automatically used by the system to simulate the effects of changing ventilator settings. The results of these simulations are then used by the system to calculate the clinical benefit of these changes. This is performed by automatically balancing the competing goals of mechanical ventilation, using a decision theoretic approach [13]. For example, an increased inspiratory volume will result in model simulations which reduce acidosis of the blood, while increasing lung pressure detrimentally. Appropriate ventilator settings therefore imply a balance between the preferred value of pH, weighed against the preferred value of lung pressure. Similar balances exist for the correct level of oxygenation, or the correct level of ventilation support when the patient is breathing spontaneously. The system weighs these balances, calculating a total score for the patient for any possible ventilation strategy. The system then calculates advice to change ventilator settings so as to improve this score. This combination of a detailed description of the individual patient’s physiology—a digital twin—along with decision theoretic models of clinical preference, is novel when compared to other ventilator advisory systems. The BC functions as an “open loop” system. This means that the advice provided by the system is presented to the clinician. Ventilator settings are then changed by the clinician, and the patient’s response to ventilator changes is used by the system to re-tune the models and repeat the process of generating new advice. In this way, the system learns from patient response to ventilator changes. In the intervention arm, the BC provides advice on the following ventilator settings: inspired oxygen fraction (FIO_2_), positive end expiratory pressure (PEEP), tidal volume (VT) or pressure level, respiratory rate (RR), and pressure support (PS), with guidance provided to maintain I:E ratios in mandatory modes. In addition, the BC provides advice on the need for extra measurements, to switch ventilator mode or to initiate spontaneous breathing tests. All other settings and therapy were decided by the attending physician. Further details on advice calculation are provided in the ESM (ESM S.2).

In the control arm, the clinical team managed weaning from mechanical ventilation as per local clinical practice. Standardisation of the control arm was outside the scope of this study. The BC did not provide advice in this arm but was used to collect data.

### Measurements

The BC was attached to the patient via a tube placed in the respiratory circuit and an additional pulse oximeter. The BC was also connected to the ventilator via a RS-232 serial cable. For all patients recruited to the study, data was collected using the BC for respiratory flows, pressures, volumetric capnography, indirect calorimetry and pulse oximetry. Values of these measurements were averaged over each minute. These represent unique, complex, datasets over the whole duration of mechanical ventilation, a description of which is included in the ESM (S.5), including illustration of a sample data set (Fig. S.5.1). Arterial blood gas samples were taken according to clinical need, supplemented by advice from the BC, and entered manually into the BC by bedside clinical nurses. In the intervention arm, the BC collected data describing the use of the system, including the presentation and use of advice.

In addition, clinical data were recorded on a daily basis using an electronic case report form system (SMART-TRIAL® Copenhagen). These data included those required for analysis of demographics and secondary outcomes. A full list of all data fields is provided in the ESM (ESM S.6).

### Data analysis and statistics

The study was powered to determine a 2-day or 30% reduction in the duration of mechanical ventilation from recruitment of 137 patients in each arm, as described previously [17]. As described in the results, the study did not reach completion and statistical results are likely to be underpowered. To maintain the integrity of the study, data was analysed for all primary and secondary outcomes ([17] and ESM S.3).

All data describing ventilator settings and physiological measurements, were averaged per patient over the relevant ventilator duration, with statistical analysis performed on these averages. For example, FIO_2_ was averaged over the complete duration of ventilation, whereas measures of rapid shallow breathing index were averaged only for support ventilator modes. Time in mandatory or support modes was calculated as the cumulative time in the mode expressed as a percentage of ventilation duration, as such accounting for repeated switching between modes. Time from mandatory mode to support mode, was calculated as the time until the first application of pressure support lasting > 30 min. This was defined to account for failed attempts to switch to pressure support mode. Initiation (Fig. S.2.5) and successful completion (Fig. S.2.6) of spontaneous breathing tests were recorded automatically by the BC. Mobilization times, and the use of other therapy was recorded daily in the electronic case reports. The number of ventilator changes was automatically counted by the BC and averaged over the relevant ventilation period depending on ventilator mode. Average duration outside physiological limits was calculated as the minutes outside the physiological limit expressed as a percentage of the relevant ventilation period.

Statistical analysis was performed with SPSS (SPSS Statistics 22.0; IBM, Armonk, NY). Normality was assessed with Quantile–Quantile-plots and Shapiro–Wilk test. Descriptive statistics are reported as mean ± SD or median (interquartile range) for normally and nonnormally distributed data, respectively. Unpaired t-test or Man-Whitney U test was used to compare values between arms for all continuous variables depending upon normality of data. Patient counts (mortality, prolonged intubation, tracheostomy and adverse events) were compared using Fisher’s exact test, when comparing the number of patients, and Man-Whitney U test, when comparing the median number of events per patient. Kaplan Meier analysis was performed for cumulative survival and probability of not reaching successful extubation, with significance calculated using the log-rank test. P-values lower than 0.05 were considered statistically significant.

## Results

**Fig. 1:**
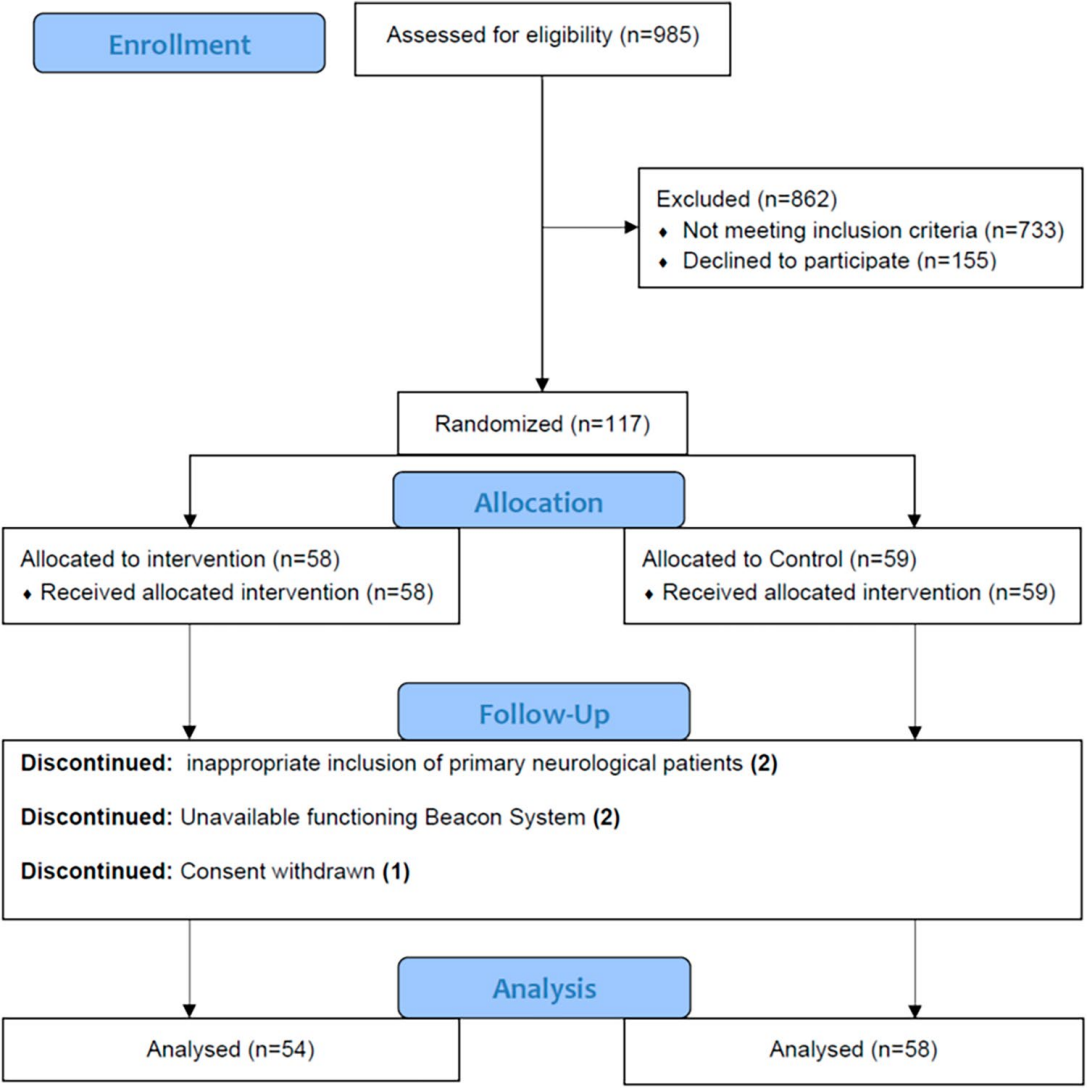
CONSORT flow diagram

The study commenced on 26/09/2017 and was completed on 17/03/2020. Between this period, a total of 985 patients were screened, 252 patients met inclusion criteria with 135 patients declining as illustrated in Fig. 1. Sites recruited 57 (CW), 31(NM), 27 (WM), 1(NN) and 1(LU) patients, respectively. Low recruitment at NN and LU was due to site initiation shortly before the onset of the COVID-19 pandemic and study termination. A total of 112 patients were included for analysis following investigator withdrawal of 5 patients due to inappropriate inclusion (2), lack of functioning BC (2) and withdrawal of family consent (1). Of these, 54 patients were in the control arm and 58 patients in the intervention arm. The total number of enrolled patients were significantly less than the predetermined sample size [17] due to a lower number of patients meeting inclusion criteria and the need to stop early due to the COVID-19 pandemic, as addressed further in the discussion.

Patient demographics and clinical characteristics are presented in Table 1. There were no significant differences between intervention and control arms, aside from an increase in intubation to maintain airway patency in the intervention arm.

**Table 1:** Patient demographics and clinical characteristics

Characteristics	BEACON Caresystem arm (BC-intervention)	Control arm (Routine Care)	p-value
No of patients	54	58	
Age in years—mean ± SD	60 ± 18	59 ± 16	0.760
Male—% within group	67	72	0.437
Female—% within group	33	28	0.437
Body Mass Index—median (IQR)	25.4 (5.7)	26.0 (5.6)	0.596
Physiological Severity Scores			
Sequential organ failure assessment (SOFA) score—median (IQR)	8 (5.2)	7 (4.0)	0.138
APACHE II score—median (IQR)	19.5 (11)	17 (9.2)	0.246
SAPS II score—mean ± SD	48.9 ± 18.3	45.1 ± 18.2	0.268
Type of admission—n			
Elective	7	5	0.436
Emergency	47	53	0.436
Reasons for admission—n/% within group			
Gastrointestinal	9 /17	8/14	0.436
Burns	7/13	8/14	0.560
Trauma and Orthopaedics	0/0	2/3	0.266
Pneumonia	15/28	13/22	0.331
Other causes of respiratory failure	8/15	7/12	0.440
Self harm/poisoning	4/7	4/9	0.601
Other diagnoses	11/20	16/28	0.252
Advanced organ support			
Continuous renal replacement therapy. Number of patients—(n)/ days per patient median (IQR)	20 / 0(3)	15/ 0(1)	0.142/0.186
Duration of mechanical ventilation at randomization, hours—median (IQR)	47.3 (56.6)	47.0 (34.3)	0.606
Reasons for intubation—n			
Inability to maintain airway patency	41	32	**0.017**
Inability to protect airway against aspiration	27	23	0.257
Failure to ventilate	23	28	0.704
Failure to oxygenate	29	32	0.557
Anticipation of deterioration in disease process leading to respiratory failure	37	37	0.549

Bold values indicate statistical significance, p<0.05

**Fig. 2:**
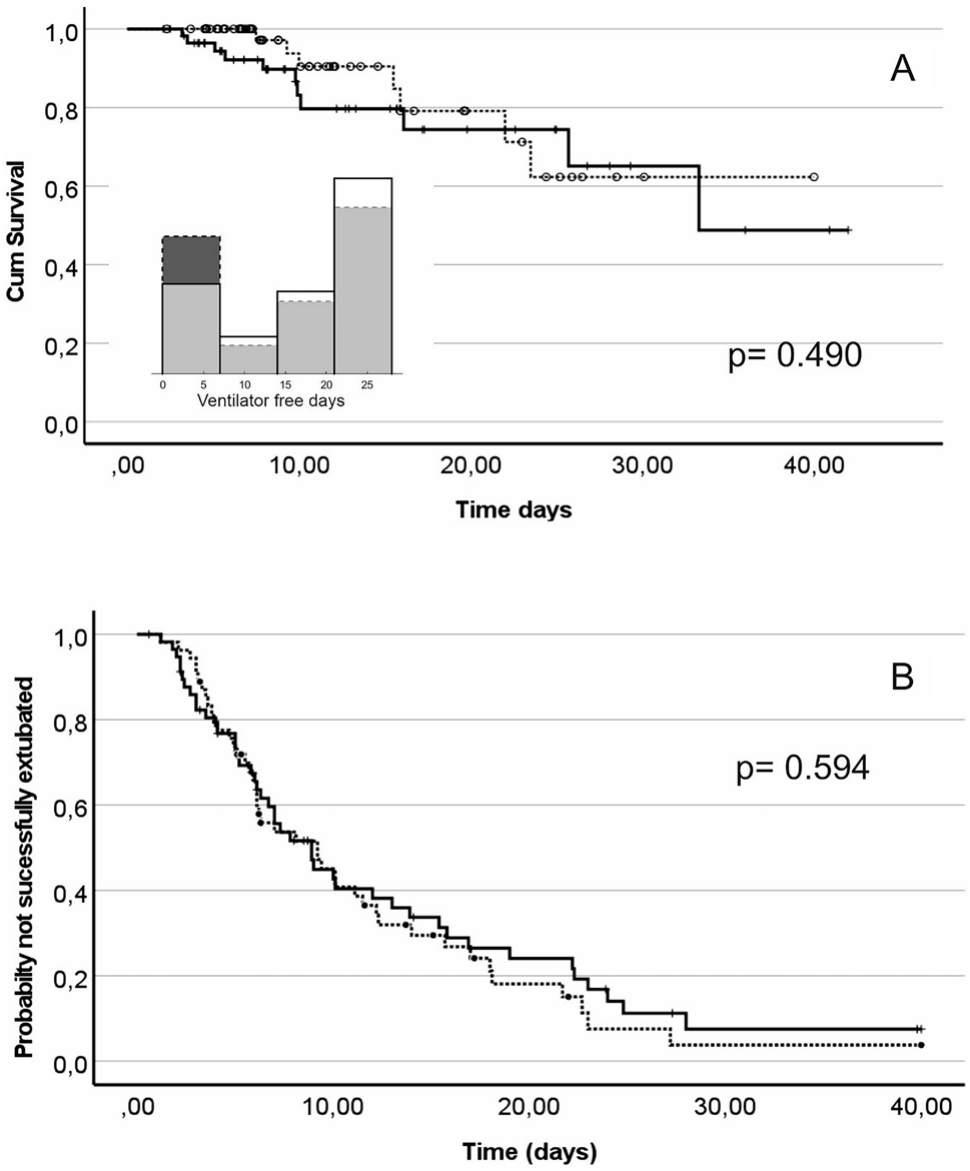
Kaplan Meier plots of cumulative survival (**A**) and the probability of not reaching successful extubation (**B**). Dashed line intervention arm. Solid line controlled arm. Inset on figure A illustrates the probability density plot for ventilator free days, for the control arm (black, dashed edged) and the intervention arm (white, solid edge). Overlap is shown in grey. p-values are for log-rank test

**Fig. 3:**
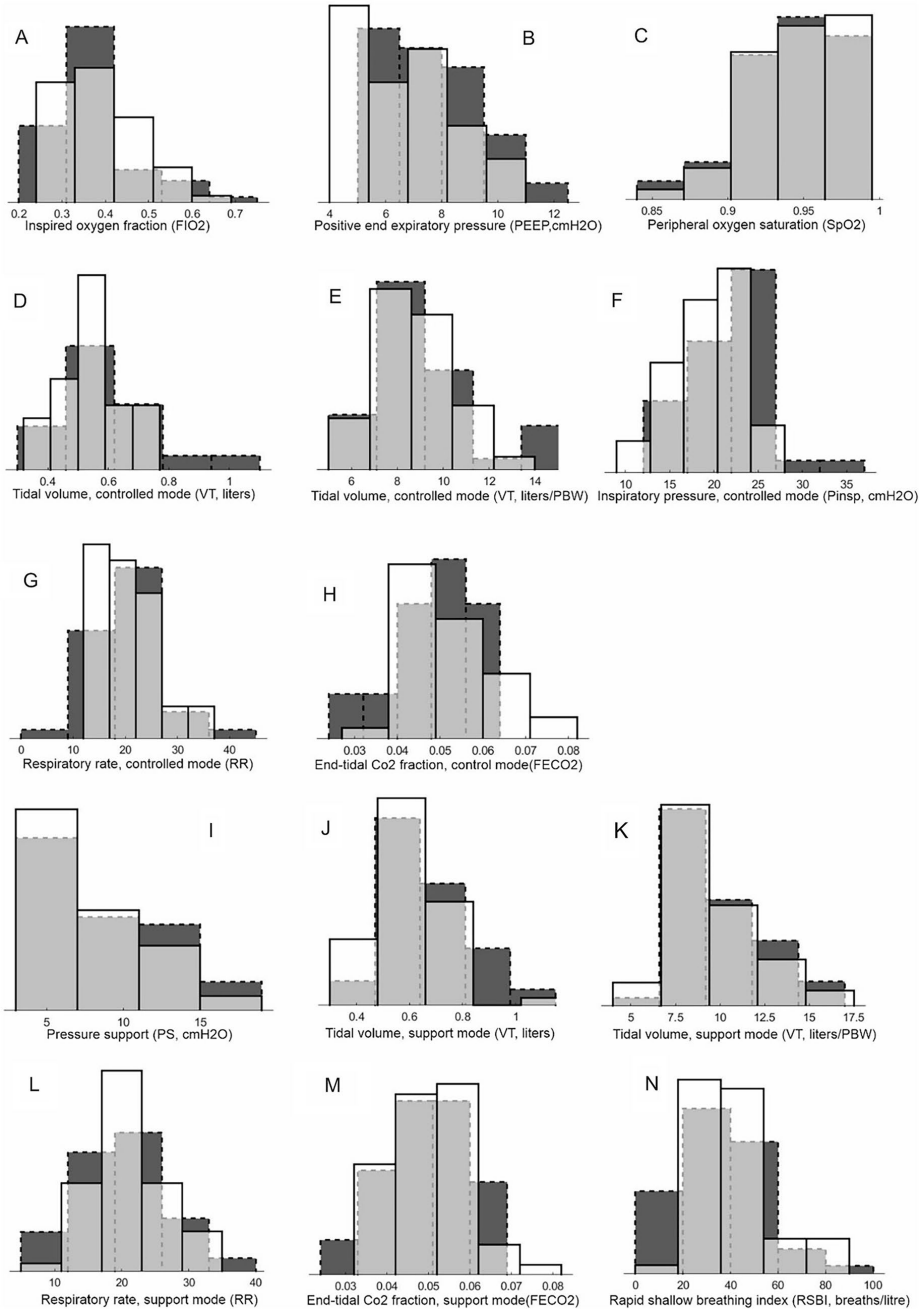
Probability density plots for the control arm (black, dashed edged) and the intervention arm (white, solid edge). Overlap is shown in grey, y-axis is probability

Study results are presented in Table 2, Fig. 2 and 3. No significant differences were seen in the durations of ventilation (Table 2) or in Kaplan Meier analysis of time to successful extubation (Fig. 2B) or cumulative survival (Fig. 2A). Intervention arm patients had lower rates of adverse event for all variables (p = 0.016), significantly fewer hypoxaemic events (p = 0.008), and a tendency to reduced adverse events for every measurement except for the presence of pneumothorax, with a single patient case in the intervention arm. Values of tidal volumes were significantly lower in the intervention arm both for mandatory modes (p = 0.042) and in pressure support mode (p = 0.041)) (Table 2, Fig. 3D,G), but not significantly so when adjusted for predicted body weight (PBW) (Table 2). In mandatory modes of ventilation, lower absolute values of VT were due to elimination of the highest values (Fig. 3D) above 0.8 l. Median values of oxygenation (SpO_2_) were similar between arms (Table 2, Fig. 3C) with similar FIO_2_ levels (Table 2, Fig. 3A) but with significantly lower values of PEEP levels in the intervention arm (p = 0.030, Fig. 3B). Values of peak inspiratory pressure (Table 2, Fig. 3F) and pressure support (rable 2, Fig. 3I) were reduced but at the boarder of statistical significance (p = 0.104, p = 0.093, respectively). No differences were seen for time in ventilator mode, time to initiation or successful SBT, or other therapy. Ventilator settings were adjusted more often in the intervention arm, with only FIO_2_ adjustments significantly so (p < 0.001). Advice was followed 88% of occasions within a 2 h window of advice presentation. For 9% of advice no change in ventilator settings was made in the 2 h window. For 3% of advice, an adjustment was made in the opposite direction to advice in either all or some of the ventilator settings (see full analysis in ESM S.10). No significant differences were seen in time spent outside physiological limits.

**Table 2:** Study results

	BEACON Caresystem arm (BC-intervention) n = 54	Control arm (Routine Care) n = 58	p-value	CI of mean difference
Duration of ventilation				
Duration of mechanical ventilation to successful extubation, days—median (IQR)	10.1 (9.5)	9.5 (10.9)	0.773	
Duration of mechanical ventilation to successful extubation following randomization, days—median (IQR)	6.3 (9.2)	7.0 (10.6)	0.798	
Time to first extubation/first disconnection from mechanical ventilation, days—median (IQR)	4.3 (6.7)	3.2 (7.1)	0.263	
Number of intubation free days at 28 days, days [18]—median (IQR)	19.4(13.5)	19.1(22.2)	0.423	
Prolonged intubation and survival				
Mortality—n/% within group	7 /13	11/19	0.447	
Prolonged intubation, > 21 days—n/%within group	9 /17	9 /16	1.0	
Length of ICU stay—median (IQR)	14 (14)	15 (16)	0.998	
Tracheostomy—n/% within group	11/20	15/26	0.505	
Adverse events (num. patients presenting/total num. events in all patients for duration of ventilation) (p-values on num. patients/median num. events per patient for duration of ventilation)				
Reintubation	7/7	10/13	0.604/0.469	
Acidemia, arterial pH < 7.2	5/6	10 /10	0.273/0.236	
Alkalaemia, arterial pH > 7.5	13/17	19/54	0.405/0.175	
Hypoxaemia, arterial SO2 < 90% or PO2 < 8.0 kPa	16/44	33/101	**0.005/0.008**	
Pneumothorax	1/3	0/0	0.482/0.300	
Self Extubation	1/1	2/2	1.000/1.000	
Composite side effects, Median per patient (IQR)	0.0 (3.0)	2.0 (4.0)	**0.016**	
Ventilator settings and physiological state				
All modes				
FIO2 level set—median (IQR)	0.36 (0.13)	0.35 (0.10)	0.555	
PEEP level set, cmH2O -– median (IQR)	6.5 (3.0)	7.4 (2.7)	**0.030**	
Mandatory modes				
VT measured, l—median (IQR)	0.53 (0.11)	0.60 (0.15)	**0.042**	
VT measure, l/kg PBW—mean ± SD	8.6 ± 1.7	9.2 ± 2.4	0.197	-0.4 1.7
Inspiratory pressure, Pinsp, cmH2O—mean ± SD	19.5 ± 3.8	21.1 ± 4.5	0.104	-0.3 3.6
Pinsp-PEEP, cmH2O—mean ± SD	12.0 ± 3.5	12.5 ± 4.0	0.611	-1.2 2.3
RR measured, min^−1^– median (IQR)	19.3 (7.1)	18.4 (5.8)	0.875	
FECO2, kPa—mean ± SD	5.1 ± 1.0	4.9 ± 0.9	0.264	-0.7 0.2
SpO2, %– median (IQR)	95.7 (4.7)	95.1 (4.7)	0.991	
Pressure support				
PS level set, cmH2O—median (IQR)	6.6 (6.4)	8.7 (6.1)	0.093	
VT measured, l—median (IQR)	0.58 (0.17)	0.63 (0.24)	**0.041**	
VT measured, l/kg PBW—median (IQR)	8.9 (2.5)	9.3 (2.9)	0.232	
RR measured, min^−1^—mean ± SD	20.8 ± 5.7	20.2 ± 6.4	0.638	-2.9 1.8
FECO2, kPa mean ± SD	5.0 ± 0.9	4.9 ± 1.0	0.791	-0.4 0.3
RSBI, ml/ min^−1^—mean ± SD	40.6 ± 17.3	35.5 ± 18.3	0.159	-12.0 2.0
SpO2, %—median (IQR)	95.7 (4.1)	93.9 (4.6)	0.116	
Patient flow				
Time in mandatory mode, %—median (IQR)	25.8 (32.7)	35.5 (72.5)	0.311	
Time in support mode, %—median (IQR)	70.7 (53.4)	64.5 (79.4)	0.262	
Time from mandatory mode to support mode, days—median (IQR)	0.07 (2.2)	0.16 (1.9)	0.866	
Time from support mode to successful extubation, days—median (IQR)	6.20 (7.2)	6.08 (11.1)	0.700	
Time from randomization to first SBT, days - median (IQR)	2.29 (6.8)	3.44 (7.4)	0.275	
Time from randomization to first successful SBT, days—median (IQR)	3.24 (7.5)	4.00 (10.0)	0.374	
Time to first mobilisation, days—median (IQR)	6.5 (6.5)	6.6 (10.8)	0.443	
Link to other therapy				
Duration sedated, %—mean ± SD	52.5 ± 26.9	51.9 ± 24.9	0.911	-10.3 9.2
Inotrope free days at 28 days, days—median (IQR)	26.6 (5.0)	26.1 (8.4)	0.324	
Neuromuscular blocking agents, num patients used/% within group	8 /15	11 /21	0.621	
Use of system—average number vent changes per day				
FIO2, n—median (IQR)	9.6 (7.0)	4.5 (3.5)	** < 0.001**	
PEEP, n—median (IQR)	0.9 (1.6)	0.9 (1.8)	0.243	
Pinsp, mandatory mode, n—median (IQR)	0.7 (4.6)	0.0 (1.7)	0.102	
RR, mandatory mode, n—median (IQR)	3.1 (9.9)	2.0 (4.9)	0.207	
PS, PS mode, n—median (IQR)	4.9 (6.5)	3.0 (8.2)	0.144	
Average duration outside physiological limits				
Time SpO2 < 88%, %—median (IQR)	3.0 (7.5)	4.8 (11.8)	0.194	
Time FECO2 > 7 kPa, %—median (IQR)	0.1 (2.8)	0.0 (0.6)	0.173	
Time VT > 8 ml/kg PBW, %—median (IQR)	15.7 (27.9)	15.6 (39.5)	0.455	
Time RR < 12 min^−1^, PS mode, %—median (IQR)	1.6 (9.2)	2.0 (15.8)	0.933	
Time RSBI > 100 ml/ min^−1^, PS mode only, %—median (IQR)	0.0 (1.0)	0.0 (0.3)	0.174	

Bold values indicate statistical significance, p<0.05

## Discussion

This is the first study to evaluate the BEACON Caresystem in comparison to routine care, over the complete duration of mechanical ventilation. Originally powered to investigate whether application of the system reduced duration of mechanical ventilation, the study was suspended and then terminated during the COVID-19 pandemic, preventing full recruitment.

No significant difference was seen in the duration of ventilation, the primary outcome. This lack of significance may be due to the function of the system, the underpowered nature of the study or the multi-factorial nature of intensive care, where ventilator duration may be due to multiple factors. Despite this, the application of advice appeared to have several significant benefits. Patients on the intervention arm had significantly lower incidence of adverse events, primarily hypoxaemia, this being despite similar FIO_2_ levels and significantly lower values of PEEP (p = 0.030). Treatment arm patients also had significantly lower levels of measured tidal volume in both mandatory (p = 0.042) and support modes (p = 0.041) but not when adjusted for predicted body weight. Median inspiratory pressure (Pinsp) (mandatory mode) and pressure support levels tended to be lower in the intervention arm but without statistical significance (0.104, 0.093, respectively). Lower tidal volumes were obtained without significant increases in end tidal levels of carbon dioxide (FECO_2_), respiratory rate or, for pressure support, the level of rapid shallow breathing index. The changes are consistent with those seen in prospective observational studies applying the advice of the BC in short durations of 4–8 h [14, 15]. For patients in mandatory mode ventilation, previous application of BC advice resulted in significant reduction in tidal volume [14]. For patients on pressure support, previous results showed significantly reduced values of pressure support [14, 15] but without dangerously high levels of rapid shallow breathing index [14, 15], or, where esophageal pressure measurements were available, without indicating diaphragm fatigue [15]. For all modes advice was previously shown to result in FIO_2_ values of about 0.35 to 0.40, consistent with both study arms reported here, and without abnormal values of SpO_2_. In addition, advice was shown to eliminate the adverse effects of passive inflation seen in high levels of pressure support [15].

It has recently been argued that unnecessary variability in therapeutic intervention, and the lack of replicable decisions, prevents the assessment of changes in therapeutic strategy [19]. Decision support systems, such as the BC, will consistently provide the same therapeutic advice for identical physiological situations and this is therefore an inherent strength of such systems. It is therefore interesting to understand whether application of the BC advice reduced variability. Although inconclusive, some indication for reduction of variability can be seen here. Median values of the number of intubation free days was similar between arms, but the inter-quartile range was higher for usual care. In addition, greater variability could be seen in values of tidal volume and peak pressures for patients in usual care.

Unlike previous studies [14, 15], the BEACON Caresystem was used in this study by the bedside nurse rather than the research team, with the studies performed in institutions where specialist nurses often adjust ventilator settings. Such studies are extremely challenging. They require establishing super-user groups, continuous training in use of the technology across a large user group, and round the clock technical support. Unlike commonly applied off-line training with mannequins and simulation models, evaluation of the BC in this RCT did not include periodic debriefing and detailed review of the systems advice. Such debriefing would have introduced bias into study results, either through adaptation of usual care or modification of the system’s function. Systems build on physiological models of the individual patient, such as the BC, are intended to promote learning at the bedside through understanding the patient’s physiological state and the necessary clinical trade-offs of therapy. Adaptation of clinical practice, and or the systems function, should therefore be a natural part of integration of such a system into daily practice. This is at odds with typical RCT design such as applied in this study, where blinding to the therapeutic effect is necessary. New study designs such as the stepped wedge cluster randomized control trial, may be more suitable to evaluation of decision support tools, allowing scientific rigor combined with learning during the study period between cluster implementation [20].

Despite the challenging nature of this study, the BC advice was applied on 88% of occasions and resulted in more frequent adjustment of ventilator settings, significantly so for FIO_2_. Situations where advice was not applied were primarily due to periods where ventilator settings were not adjusted at all within the 2 h window. A total of 9% of advice was not acted upon, meaning that neither these, nor other ventilator changes were made within a 2-h window. This is, perhaps, to be expected in a situation where the system is applied 24 h a day, and where there may be long periods where advice might have gone unnoticed, or ventilator management was not at the fore. It is clear, that it is such situations where closed-loop application might be beneficial. For 3% of advice, a ventilator adjustment was made in the opposite direction to that advised either for all settings or a subset. Such cases require individual assessment to understand differences in the function of the system and clinical opinion, and it is these cases which provide the opportunity to learn about discrepancies between system function and clinical practice, part of a learning healthcare system [19]. This was often the case for complex advice involving suggestions of more than one setting adjustment, for example tidal volume and respiratory frequency, and where setting of only one of these—even in the correct direction—was counted in the analysis as non-compliance (see ESM S.10).

Other studies have investigated the use of open-loop advice based on rules [21], or rules combined with neural networks [9, 22]. The former showed improvement in morbidity scores in 200 patients with ARDS [21] but did not show improvement in ventilation duration. The latter is the commercial VentAssist system, which has largely been evaluated in studies assessing clinical agreement to advice on pressure support [22] with a single study illustrating improved work of breathing in a small, randomized trial of 44 patients [9]. The most widely applied and evaluated routine commercial tools—SmartCare (Dräger Medical) and Intelli-Vent ASV or its predecessor ASV (Hamilton Medical), apply closed-loop automation rather than open loop advice. SmartCare has been evaluated in several RCT’s. Two have shown significant reduction in weaning duration in multicenter studies of 144 [7] and 92 patients [23]. A single study of 102 patients showed no reduction in ventilator duration [24] but improvement in time to reach conditions suitable for ventilator separation in patients who were not as severely ill. The largest study to date, in 300 patients, showed no significant reduction in length of duration over all patients, but significant reduction in a subset of 132 patients having undergone cardiac surgery [25]. ASV and Intelli-Vent ASV control the patient through all phases of ventilation, with only IntelliVent ASV controlling FIO_2_ and PEEP. Early studies showed significant reductions in weaning time for fast-track cardiac patients (8–15 h ventilation) in small studies of about 40–50 patients [26, 27]. A single large study has shown significant reduction in ventilator time in 229 patients ventilated longer than 24 h [28], with a further showing reduced ventilator time in 97 COPD patients during support mode ventilation [29]. Many other studies have focused on improved physiological state in relation to time spent in optimal regions of ventilator management or workflow improvement analysis without outcomes related to ventilation duration [30–35]. The study presented in this paper represents the first evaluation of the BEACON Caresystem for application in the full duration of mechanical ventilation, and further data is required to place it in the context of these other systems.

## Limitations

This study was terminated early due to the Covid-19 pandemic. It is worth noting that the rate of consent of 44% was close to the 50% expected (see Fig. 1 in [17]]. The main contributors to lack of eligibility in the study were admissions requiring short periods of ventilation in the ICU and a larger than expected numbers of patients with primary neurological problems.

The study was a pragmatic study comparing the system to current clinical practice. As recognized in the study design [17] no limitations or standards were placed on the therapy of the control arm. As such this study compares the system against the standard of care of these institutions, and not necessarily against best practice in general. Establishing a best practice protocol is complex and would have required multiple simultaneous interventions.

Future studies are required to understand the application of the system in other patient groups and institutions to investigate whether the results presented here can be generalized. Studies are currently underway investigating the use of the system in ARDS including COVID-19 patients [36], and in a cardio-thoracic ICU setting [37].

## Conclusion

This study has investigated application of advice from the BEACON Caresystem in a general ICU population ventilated for more than 24 h. The study was terminated early, with no significant difference shown in duration of mechanical ventilation, the primary outcome. Application of advice indicated potential for fewer adverse events and improved physiological status.

## Supplementary Information

Below is the link to the electronic supplementary material.

Supplementary file1 (DOCX 2215 KB)

## Data Availability

An example of a full data set is provided in the electronic supplementary material.
