# Supplementary material for: Evaluation of decision support to wean patients from mechanical ventilation in intensive care: a prospective study reporting clinical and physiological outcomes
Source: J Clin Monit Comput. 2024 Nov 9;39(2):393–404. doi: 10.1007/s10877-024-01231-5 (PMC12049327; doi:10.1007/s10877-024-01231-5)
Supplement: Supplementary file 1 — Supplementary file1 (DOCX 2215 KB) [file 10877_2024_1231_MOESM1_ESM.docx]

**Supplementary Material**

Index of supplementary material

S.1) Study procedure

S.2) Structure and Function of Beacon Caresystem

S.3) Study outcomes

S.4) Inclusion/Exclusion criteria

S.5) Data collection from the Beacon Caresystem

S.6) List of variables collected on eCRF

S.7) Strategy for site initiation, training and support of clinical personnel

S.8) Research governance

S.9) Data on cause of lack of eligibility

S.10) Evaluation of advice use

S.11) Post-hoc analysis of high tidal volumes in control mode, and high SpO_2_ values.

S.12) References

S.13) List of investigators and acknowledgements

**S.1) Study procedure, summarized from [1]**

Patients were randomised to either to the intervention group where advice is provided by the BEACON Caresystem, or to the control arm where routine care was provided. Block randomisation was applied to ensure equal distribution between control and intervention for each of the following groups: Surgical or medical AICU admission; presence of absence of chronic obstructive pulmonary disease and primary thoracic or non-thoracic medical diagnosis.

In the intervention arm of the trial, advice was provided by the Beacon Caresystem (BC). In the control arm, BC advice was disabled, but data were still collected by the BC. During intervention, the BC provided a number of different forms of advice in addition to ventilator settings. These were: to perform an arterial blood gas (ABG) or a procedure to estimate gas exchange parameters (ALPE); to suggest change in ventilation mode from control to support on detection of substantial spontaneous breathing or for support to control if arterial pH falls below a threshold; to monitor pressure support levels and identify clinical thresholds for SBT prompting the user to consult an extubation checklist if thresholds were maintained for a fixed duration. The protocol ended with transfer or discharge from AICU or death.

Figure S.1.1 Consolidated Standards of Reporting Trials flow diagram for protocol. MV, mechanical ventilation. This flowchart is reproduced from Vizcaychipi et al [1], with the sub-flowchart included in point S.2 of this ESM.

**S.2) Structure and Function of Beacon Caresystem**

This section describes the structure of the BC in terms of the mathematical models included in the system, and the function of the system in providing advice on ventilator settings, mode changes, requesting arterial blood gasses, and guiding SBT evaluation. Examples are provided of the screen displays and the bedside use of these. Much of this supplementary material has been provided previously by the some of the authors of this work [1,2,3].

Mathematical models included in the CDSS

Figure S.2.1 illustrates the structure of the mathematical models with a full description of model formulation and evaluation published previously [4]. The system includes mathematical models of pulmonary gas exchange; respiratory mechanics; acid-base chemistry of blood, interstitial fluid, tissues and cerebral spinal fluid; respiratory drive and ventilation. In addition, the models include the effects of PEEP on gas exchange, pulmonary mechanics, and ventilation. All models are tuned to the individual patient’s physiological status through measurement of respiratory gas flows and pressures; calorimetry and capnography measurements of respiratory gas fractions of O_2_ and CO_2_, and subsequent calculation of oxygen utilisation (VO_2_) and carbon dioxide production (VCO_2_); pulse oximetry measurement of arterial oxygen saturation; and arterial blood measurements of acid-base, oxygenation and haemoglobin fractions. The model of pulmonary gas exchange is tuned to the appropriate matching of ventilation and perfusion to account for O_2_ and CO_2_ differences between arterial and end tidal gas values. To do so an arterial blood gas (ABG) is required on system start up. In some patients, the system also requires modification of FIO_2_ to two levels for 2-5 minutes at each level to tune the pulmonary gas exchange model to the patient [5,6,7].

The respiratory mechanics model is tuned to dynamic compliance. The model of acid-base chemistry of the blood is tuned to measured values of arterial pH, PCO_2_, PO_2_, and SO_2_, and haemoglobin concentration, with the acid-base chemistry of the cerebrospinal fluid (CSF) regulated to arterial bicarbonate values to account for chemical changes in respiratory drive. The respiratory drive model is tuned to describe the relationship between alveolar ventilation (VA) and arterial acid-base and oxygenation status. The ventilation model is tuned to the measured serial dead space (Vd) to allow calculation of alveolar ventilation. A series of algorithms are present in the system to re-tune the models as new measurements present, or if the patient condition changes. These models are used to simulate the effect of changes in ventilator settings, with the system generating advice based upon simulated values. The mathematical models describing the response to changes in PEEP on pulmonary gas exchange and mechanics has been described in previous supplementary material [2].

Figure S.2.1: Mathematical models included in the system, modified from [4]. Mathematical models are indicated by dashed boxes with the exception of the acid-base model of blood, which is illustrated in each blood compartment as used. Measured input values are indicated by solid boxes.

Use of the Beacon Caresystem

Figure S.2.2 illustrates the use of the BC in the intervention arm, as published previously [1]. The primary functionality of the system is to provide advice on ventilator settings. In addition, the system provides advice on the need for an arterial blood gas (ABG) or a short procedure to evaluate gas exchange (ALPE [5,6]), whether the ventilator advice is out of bounds, advice on mode change and advice that SBT conditions have been initiated and maintained such that extubation criteria can be considered. These are considered here in turn.

Figure S.2.2. Flow chart of intervention arm. ABG- arterial blood gas, ALPE – automatic lung parameter estimator, CRF – clincal research form, SBT – spontaneous breathing trial. This flowchart is reproduced from Vizcaychipi et al [1],

*Ventilator advice*

Advice is calculated by the system using the mathematical models, described above, tuned to the specific patient measurements so as to be individualized, along with a set of clinical preference functions which quantify and balance the negative outcomes of mechanical ventilation in a decision theoretic approach [8]. Model tuning, the clinical balances and the resulting advice screen are illustrated in figure S.2.3 as published previously [9]. The balances are: between over and under oxygenation; the balance between the risk of ventilator induced lung injury due to high pressures, respiratory rate or tidal volumes balanced against the risk of acidosis with insufficient CO_2_ expiration; and for pressure support, the balance between the risk of respiratory muscle atrophy with over support, and a stressful situation for the patient with excessive work of breathing, for under support. Advice is provided in steps moving the patient toward the ventilator settings which the system calculates as most optimal for the patient at that time. Following change of ventilator settings by the clinician, a period of 5–20 min is waited by the system to ensure the full effects of the ventilator changes are complete, and new advice then generated. If system calculations show no further ventilator changes can improve overall patient state then no further advice is generated until the patient state changes. In this way, the frequency of advice depends upon the stability of the patient and the difference between current and optimal ventilator settings. Physiological models are re-tuned as the patient state changes and on measured response to changes in ventilator settings.

Figure S.2.3 – Beacon Caresystem structure and advice screen, see [9] for details. Modified from [9].

Advice is presented in several ways, as illustrated in figure S.2.4. In its simplest form, advice is presented as the current and suggested ventilator settings. If requested, the user can view the clinical trade-offs. The white “simulated” settings also allow the user to simulate the response of different settings. For the more expert user, the values of current, simulated and advised physiological variables are also displayed. These screens are intended to let the user decide on the complexity of the system depending upon their own expertise and wishes.

Figure S.2.4 – Beacon Caresystem advice presentation illustrating the different complexity of advice presentation selected by the user.

*Advice on arterial blood gas ABG, and a procedure to estimate pulmonary gas exchange (ALPE)*

The patient specific mathematical model, tuned to fit measurements are used within the system to simulate current values of continuously measured variables, e.g. SpO_2_, end-tidal CO_2_ fraction, or in support ventilator modes, respiratory frequency or tidal volume. This simulation occurs continuously throughout the management of the patient. In situations where the mathematical model simulations cannot accurately describe continuous measurements, then the models no longer reflect the patient conditions and re-tuning of the models is required. In this situation, the BC requests either an ABG, or a short procedure varying FIO_2_ to assess pulmonary gas exchange (ALPE), as described and evaluated previously [5,6]. In this way an ABG is taken when the current measurements cannot be explained by the mathematical model description of the patient, rather than at fixed time periods, representing a rational strategy toward measurement in the ICU.

*Advice to change ventilator mode*

This occurs if the patient is in a controlled ventilator mode and the system identifies a total respiratory frequency or a total minute ventilation that is significantly greater than that set. In this case the system provides a message to the user suggesting changing to support mode. Similarly, if the patient is in support mode and the model simulated pH is very low then the system provides a message asking the user to consider either control mode or reduction of anaesthesia. In ventilator modes which provide both mandatory and spontaneous breaths, the system provides advice on support or control ventilator settings depending upon the pattern of patient ventilation, and whether the patient is currently in a period with substantial spontaneous breathing activity.

*SBT setting and review, and extubation check-list*

For patients ventilated in pressure support mode with values of PEEP and PS below threshold values illustrated in figure S.2.5, the system provides a counter on the screen which counts down from 30 minutes, and as such indicates that the patient has been within ventilator settings consistent with an SBT and stable with regard other respiratory measurements, for this duration. The default cut-off values for these other variables are illustrated in figure S.2.5.

Figure S.2.5 – User interface illustrating variables used to time the SBT counter of the system.

At any point when the counter is running, the user can view the screen illustrated in figure S.2.6. This indicates which variables are within threshold, their values, and if all have been maintained at these levels for the required duration.

Figure S.2.6 – User interface illustrating status of variables used to track the stability of patients over time in relation to the SBT counter. Taken from the simulator mode of the BC to illustrate the case.

When all variables illustrated in figure S.2.6 are within range for the expected duration, the system allows checking of an extubation checklist illustrated in figure S.2.7. Neither the SBT screens S.2.5 and S.2.6, nor the extubation checklist advise on an active SBT test or extubation.

Figure S.2.7a – Extubation checklist, top part of vertical scrolling window.

Figure S.2.7b – Extubation checklist, bottom part of vertical scrolling window.

**S.3) Study outcomes**

Primary outcome

Duration of mechanical ventilation to successful extubation is defined as the time from the start of mechanical ventilation until successful extubation. Start of mechanical ventilation is defined as either the time of intubation in the ICU, or the time of admission to the ICU following previous intubation for surgery. Successful extubation is defined as >48 hours of unassisted spontaneous breathing after extubation. Unassisted spontaneous breathing is defined here to include supplementary oxygen and support with high-flow nasal catheters. Patients with non-invasive mask ventilation are considered to be assisted.

Secondary outcomes

The secondary objectives of the trial are to compare the following variables in the routine care group and Beacon Caresystem group:

Duration of mechanical ventilation to successful extubation following randomisation defined as the time from randomisation and until successful extubation, with successful extubation defined as >48 hours of unassisted spontaneous breathing after extubation.

Time from control mode to support mode defined as the time following randomisation, from initiation

of control modes of ventilation and until initiation of support modes of ventilation.

Time from support mode to successful extubation defined as the time following randomisation, from

initiation of support modes of ventilation and until successful extubation.

Number of changes in ventilator settings per day defined as the daily registered number of changes to ventilator settings from randomisation until successful extubation.

Time to first SBT defined as the time from randomization to the first performed SBT.

Time to first successful SBT defined as the time from randomisation to the first successful SBT.

Time to first extubation defined as the time from randomisation to the first extubation attempt.

Time to first disconnection from mechanical ventilation defined as the time from randomisation to

the point at which mechanical ventilation is first discontinued.

Percentage of time in control mode ventilation defined as the time from randomisation which is spent in controlled modes of mechanical ventilation in per cent of duration of mechanical ventilation.

Percentage of time in support mode ventilation defined as the time from randomisation which is

spent in support modes of mechanical ventilation in per cent of duration of mechanical ventilation.

Use of sedatives defined as the cumulative use of sedative drugs from randomisation until successful extubation.

Use of neuromuscular blockading agents defined as the cumulative use of neuromuscular blockading

agents from randomisation until successful extubation.

Number of intubation free days defined as the number of days without intubation from randomisation until successful extubation.

Number of reintubations defined as the number of reintubations following extubation from randomization until successful extubation.

Number of tracheostomies defined as the number of patients having tracheostomy performed from randomization until successful extubation or protocol end.

Number of patients on prolonged mechanical ventilation defined as the number of patients on mechanical

ventilation ongoing for more than 21 days after the start of mechanical ventilation.

Number and types of adverse events related to mechanical ventilation defined as the incidence of adverse

events directly related to mechanical ventilation.

ICU mortality defined as the mortality from randomization and until death or ICU discharge.

Length of stay defined as the duration of ICU admission from the start of mechanical ventilation and until

death or ICU discharge.

Mobilisation times defined as the time from randomization until (1) first mobilisation, for example, sitting

on the edge of the bed, standing up and marching on the spot and (2) regaining independency, for example, able to drink/eat or comb hair.

Daily patient physiological blood gas status defined as daily ABG values of SaO_2_, PaO_2_, PaCO_2_, pH from

randomisation until successful extubation.

Daily average physiological status defined as daily averages of measured values of oxygenation (SpO_2_),

end-tidal CO_2_ fraction (FECO_2_), ventilation (respiratory rate, tidal volume, anatomical dead space), pulmonary mechanics (airway pressure, respiratory system compliance) and ventilator settings as measured routinely at the bedside.

**S.4) Inclusion/Exclusion criteria**

Inclusion criteria

1. At least 24 hours of invasive mechanical ventilation.

2. Age ≥18 years.

3. Patient is mechanically ventilated in a ventilator mode, and by a ventilator, supported by the Beacon Caresystem.

4. Haemodynamically stable, where instability is defined by the presence of two or more of the following criteria: acidosis pH <7.2, poor urine output <0.5 mL/kg/h, use of vasopressors, for example, noradrenaline >25 μg/min.

Exclusion criteria

1. The absence of an arterial catheter for blood sampling.

2. Medical history of home mechanical ventilation which may lead to prolonged stay in the ICU, including long-term oxygen therapy and non-invasive ventilation not associated with sleep apnoea.

3. Clinical conditions requiring treatment with extracorporeal membrane oxygenation (ECMO), that is, an inspired oxygen of 100% for more than 24 hours.

4. Head trauma or other conditions where intracranial pressure may be elevated and tight regulation of arterial CO2 level is paramount.

5. Primary (non-overdose related) neurological patients (Glasgow Coma Score <10).

6. Severe heart failure classified by grade 4 American Heart Association guidelines.

7. End-stage liver disease.

8. Multiple ICU admissions within the medical stratification group, that is, more than one admission.

9. Corrective orthognathic surgery.

10. ICU admission following oesophageal surgery.

11. Patients with morbid obesity defined as either body mass index (BMI)>45; or BMI>35 with an Acute Physiology and Chronic Health Evaluation II (APACHEII) score on admission >24.

12. Pregnancy.

13. Mechanical ventilation initiated for more than 24 hours in other centres.

**S.5) Data collection from the Beacon Caresystem.**

The system includes a tablet computer, plus a gas module including volumetric capnography and indirect

calorimetry, with both mounted on the ventilator or a separate trolley. The patient is connected to the

system via a respiratory tube measuring flow and with side stream sampling for measurement of O_2_ and

CO_2_. The system is also connected to the ventilator data output to allow for recoding of ventilation settings and changes and ventilator mode.

The following physiologic data were collected from the measurements taken by the BC, and saved as averages over a 1 minute duration for the full duration. Data was saved as spreadsheets including 1440 rows, one per minute, for each day of ventilation.

SpO_2_ – Pulse oximetry oxygen saturation (%).

FetO_2_ – End tidal oxygen fraction.

FetCO_2_ – End tidal CO2 fration.

VO_2_ - Oxygen consumption (l/min)

VCO_2_ – Carbon dioxide production (l/min)

Rf – measured respiratory frequency.

Vt – measured tidal volume (l)

Vd – Anatomical dead space estimated from volumetric capnography (l).

Pplat – Measured plateau pressure (cmH_2_O).

Comp – Respiratory system compliance (l/cmH_2_O)

RSBI – Rapid shallow breathing index (Rf/Vt) (breaths/l)

RSBIWA – Weight adjusted RSBI (breaths/l/kg)

MV – minute volume (l/min)

VA – alveolar minute volume (l/min)

EE – energy expenditure (kcal/min)

FIO_2_Set – Ventilator set inspired oxygen fraction.

VtSet – Ventilator set tidal volume (l)

PCSet – Ventilator set pressure control level (cmH_2_O)

RfSet – Ventilator set respiratory frequency.

IESet – Ventilator set Inspiratory:Expiratory ratio

PEEPset – Ventilator set positive end expiratory pressure.

In addition the following recordings were taken from the BC to analyse use of the system advice as described in section S.9 of this document. These variables describe the state of the system at any time point, and if advice presents the values of ventilator setting for this advice. The variables recorded are

System status variables

Advice provided, awaiting response.

Patient not in steady state.

ABG request provided, awaiting response.

Typing in ABG.

Performing ALPE.

System paused, physiotherapy etc.

Unsupported ventilator mode.

Waiting, poor signal.

Systems alarm status.

Advice variables

FIO_2_ advice

Rf advice

Pressure advice

Vt advice

PEEP advice

Advice bounds exceeded

Change mode advice

In addition to values recorded on a minute by minute basis, values from arterial blood gas input into the BC were recorded, these included SaO_2_, PaO_2_, PaCO_2_ pH, BE, Hb, FMetHb, and FCOHb. Cardiac output values were either entered into the system, or estimated from IBW.

Ventilator mode – This was saved for both the mode reported by the ventilator, and that interpreted by the BC depending on the patient’s breathing pattern.

SBT status – Data was saved recording whether the conditions consistent with initiating SBT had been reached and whether these conditions had been maintained for sufficient time to qualify as SBT success.

A patient data example

A single patient example of the physiologic variables plotted over the complete duration of ventilator management is provided in figure S.5.1.

This data is that of a patient ventilated on the intervention arm for a period of 2.8 days until extubation. Plot A illustrates the ventilation mode during these days. The blue line illustrates the ventilation mode set on the ventilator, and the red that measured from the patient status. A value of 18 for the blue line and 4 on the red signifies volume control mode for the first 2.2 days. The values of 16 for the blue line, and 3 for the red signify pressure support. On all plots black stars illustrate advice from the Beacon Caresystem, which for plot A illustrates the points at which the system recommended a change to support mode from control mode.

All plots except A are color coded according to ventilator mode with magenta illustrating measurements in volume control and red pressure support. Plot B illustrates pressure. Peak pressures are plotted as magenta and dashed red lines. The solid red line and black stars illustrate PS set levels and advice during the PS period, about 5 cmH_2_0 below the Peak level, due to PEEP.

Plot C illustrates set (solid magenta) and measured tidal volume (dashed magenta and red), and system advice (black stars). For this patient, tidal volume increased substantially on switching to support mode. Plot D is analogous to plot C but for respiratory rate.

Plot E illustrates the end tidal CO_2_ levels for control and support periods, with arterial PCO_2_ levels annotated with red stars. Plot F is the RSBI index. This has little meaning for control mode period unless respiratory muscles are active.

Plot G illustrates set PEEP and PEEP advice (black stars). For this patient, PEEP was maintained at 5 cmH2O.

Plot H illustrates the respiratory system compliance as measured at the patient’s mouth. This is a variable describing the relationship between tidal volume and pressure delivered by the ventilator. Respiratory muscle activity resulting in higher tidal volume not dependent on ventilator pressure will therefore result in an increased compliance measure. For this patient compliance remained constant at about 30 ml/cmH2O for the first 1.4 days. Subsequently, it varied until the patient was switched to support mode, at which point it increased dramatically. The increase in compliance at about 1.4 days is consistent with the Beacon advice to switch to PS mode at this point, as seen by the black stars on sub-plot A and the variability in RSBI seen in sub-plot F. This advice was taken at 2.2 days.

Plots I and J illustrate the O_2_ consumption and CO_2_ production, respectively. For this patient, these remained similar and at about 0.25 l/min during control mode ventilation, increasing to about 0.35 l/min on support ventilation, probably reflecting the greater energy expenditure during support mode ventilation.

Figure K illustrates inspired oxygen fraction for control and support mode and advice. Vertical spikes to 100 FIO_2_ are frequent in all data sets and represent oxygen flushing prior to endotracheal suctioning. Figure L illustrates arterial oxygen saturation from pulse oximetry, with arterial SO_2_ levels annotated with red stars.

Figure S.5.1. Data collected from a single patient on the intervention arm. A- Blue line: ventilation mode set on ventilator, values 18 - Volume controlled, 16 – Pressure support. Red line: ventilation mode interprertation by BC, values 4-Volume controlled, 3- Pressure support. Black stars: advice to switch mode. All other plots (B-L), Magenta solid line: volume controlled mode ventilator settings; Magenta dashed line: volume controlled mode measurements; red solid line: pressure support mode settings; red dashed line: pressure support mode measurements; black stars: BC advice on ventilator settings; red stars: measurements from arterial blood gas (E-PCO_2_, L-SO_2_)

**S.6) List of variables collected on eCRF**

An electronic case report form was completed for each of the enrolled subjects, recording the eligibility criteria and consent, study stratification (surgical/medical, COPD/not COPD, medical thoracic/medical non-thoracic), study arm allocation, and demographic characteristics (Sex, age, weight, height). Also recorded were data describing the study start (date and time, duration of mechanical ventilation at the study start), admission data, such as, date of hospital admission, type of admission, date of ICU admission, admission diagnosis, date and time of intubation; reason for intubation, ARDS diagnosis – time of diagnosis, criteria and cause; and acuity scores Sequential Organ Failure Assessment (SOFA), APACHE II, SAPS II Score were captured at baseline for each participant.

Additionally, mean values of physiological parameters, such as heart rate, systolic blood pressure, diastolic blood pressure, mean arterial blood pressure (MAP); clinical procedures, administration of sedation/analgesia, muscle relaxants/vasoconstrictors/pressers were captured daily until successful extubation. Further information regarding mobilisation (First mobilisation, regaining independency), Extubation /NIV/reintubation (Extubate, endotracheal tube replaced with tracheostomy, decannulated; date and time of NIV start and end ; Reintubation (date and time of reintubation; reason for reintubation) and clinical events were collected daily from the start of the study until discharge from intensive care. Also recorded were the date and time of study end and reason for study end, ICU discharge, ICU death, patient mechanically ventilated for ≥40 days.

Listed below are the clinical procedure, medication and clinical events captured throughout the study.

Table S.6.1 - Clinical Procedures

| Airway & respiratory Procedures | Tracheostomy  LMA insertion  Fiberoptic laryngoscopy  Bronchoscopy  Bronchoalveilar Lavage (BAL)  Thoracocentesis  Proned positioning  Chest drain removal |
| --- | --- |
| Neurological Procedures | Lumbar puncture  Epidural Analgesia  Cranial Burr Hole External Ventricular Drainage  Intracranial pressure monitoring  Nerve & Muscle Biopsy  Spine immobilisation |
| Vascular & cardiac Procedures | Peripheral venous cannulation  Central Venous Catheterisation(CVC)  Peripherally inserted Central Catheterisation  Tunnelled CVC line  Intraosseous cannulation  Arterial sampling  Arterial cannulation  Pulmonary artery catheterisation (Swan-Ganz)  Pericardiocentesis  Electrical cardioversion  Temporary cardiac pacing  Intra- aortic Balloon Pump Counterpulsation |
| Gastrointestinal/Abdominal/  Genitourinary procedures | Nasograstric tube placement  Nasojejunal tube placement  Percutaneous Endoscopic Gastrostomy  Ballon tamponade in Upper GI Haemorrhage  Gastric lavage  Intra-abdominal pressure monitoring  Paracentesis  Dynamic abdominal wall closure of open abdomen  Urethral catheterisation  Peritoneal dialysis catheter placement  Haemodialysis  Bone Marrow Aspiration  Bone Marrow Biopsy  Pressure ulcer management |

| Sedation, opioids and non-opioid | Midazolam (mg/hr)  Propofol 1% (mg/hr)  Propofol 2% (mg/hr)  Clonidone (mcg/hr)  Dexmedetomidine (mcg/kg/hr)  Fentanyl (mcg/hr)  Remifentanil (mcg/kg/min)  Morphine (mg/hr)  Alfentanil (mg/hr)  Ketamine (mcg/kg/min) |
| --- | --- |
| Vasopressors/ vasoconstrictor and muscle relaxants | Noradrelaline (mcg/min)  Adrenaline (mcg/min)  Metaraminol (mg/hr)  Dobutamine (mcg/kg/min)  Dopamine (mcg/kg/min)  Hydrocortisone (mg/day)  Vasopressin (units/min)  Terlipressin (mg/hr)  Milrinone (mcg/kg/min)  Levosemendin (mcg/kg/min)  Glyceryl Trinitrate (mg/hr)  Atracurium (mg/hr)  Rocuronium (mg/hr)  Suxamethonium (mg/hr)  Vercuronium (mcg/kg/hr) |

Clinical Events

Cardiac arrest

Hypertension (SBP >150 mmHg, or DBP > 90 mmHg) >2 consecutive readings or >2 hours

Hypotension (SBP <90 mmHg, or DBP <60 mmHg) >2 consecutive readings or >2 hours

Tachycardia (HR > 110bpm) >2 consecutive readings or >2 hours

Bradycardia (HR <40 bpm) >2 consecutive readings or >2 hours

Reduction in cardiac output (CO < 4.0 L/min, or 20% reduction in CO)

Pneumothorax

Ventilator associated pneumonia (VAP) – defined by new lung infiltration of suspected infectious origin by presence of fever; purulent sputum; leukocytosis; decline in oxygenation; and occurring 48 hours after endotracheal intubation

Pulmonary embolism

Deep vein thrombosis

Fever (Temperature ≥38 °C)

Bacteraemia (confirmed by positive blood culture; resolved after completion of course of antibiotics)

Extended spectrum β-lactamase infection (confirmed by microbiology culture)

Delirium (confirmed by I-CAM positive documentation in the medical notes)

Central venous catheter - Pneumothorax

Central venous catheter– Significant haemorrhage (requiring at least 2 Units of RBCs)

Post extubation aspiration

Endotracheal tube - Unintentional Extubation

Elevated creatinine (>25% increase from baseline)

Decreased urine output (0.5mL/kg/hr) >2 consecutive readings or >2 hours

Hypoxaemia (SpO2 <90%, or <88% in COPD; or PaO2 <8.00 kPa on arterial blood sampling)

Alkalaemia (pH >7.5) – Reported in at least 2 measurements taken 20 minutes apart

Acidaemia (pH <7.2) - Reported in at least 2 measurements taken 20 minutes apart

Hypoglycaemia (<4)

Hyperglycaemia (>18)

INR increase (INR>5)

Decrease in haemoglobin value <70 (<80 past medical history of ischaemic heart disease)

Heparin induced thrombocytopenia (HIT) (HIT screen positive)

New ulcer (gastric/duodenal) diagnosis confirmed by endoscopy, during ICU admission

Norovirus infection (positive stool sample)

C.Difficile infection (positive cultures/GDH toxin)

Self-extubation

Adverse Events

All adverse events were documented and monitored until the end of the study. Adverse events included:

Pneumothorax

Hypoxaemia (SpO2<90%, or <88% in COPD; or PaO2<8.00kPa on arterial blood sampling)

Alkalaemia (pH>7.5)

Acidaemia(pH<7.2)

The following variables were collected as part of the serious adverse event reporting procedure:

Details of person making the report

Details of the study

Details of participant affected by SAE

Details of SAE (Initial and follow-up report)

Description of the event

Classification of SAE

Onset date and time

End date and time

Outcome (resolve, ongoing, died)

Action taken

Causality and expectedness ( Not related; unlikely to be related; possible related; probably related; defenitly related)

**S.7) Strategy for site initiation, training and support of clinical personnel**

The BC is an open-loop system and therefore, substantial user training was required. A standard operating procedure and easy made guideline was developed by the research team and described here. The delivery research team and nurse super-users were trained by computer engineers and BEACON team experts prior to initiation of the study. Following the sites initiation, the delivery research team conducted regular training and delivered refresher courses including at the elbow training for all new clinical incomers, allowing the system to be operational at all beds and ready to be activated as soon as patients were recruited into the study.

All sites received research and study related training, comprising Site Initiation Visit (SIV); Beacon Caresystem training; electronic Case Report Form (CRF) training (Smart-Trial, Aalborg, Denmark) and supportive information.

Site Initiation Visit (SIV)

Study organisational structure

Key contacts

Overview of the study protocol: background & rationale

Study design and outcomes

Eligibility criteria

Study interventions

Randomisation

Event reporting

Monitoring

Study aids

Investigator / Site responsibilities

Smart Trial (eCRF) – including demonstration

BEACON Caresystem training

The training program was designed to the site teams to be able to continue the keep clinical staff up skilled and confident when using BEACON Caresystem and delivering study procedures. A comprehensive training of the BEACON Caresystem was delivered to key personnel at each site (super-users) using the BEACON Caresystem – Demonstration Operator’s Manual. Two training modes were available, support mode and control mode. The first runs through a process of 5 advices while the second runs through 6 advices. Both scenarios included explanation of how to start-up, input patient demographics and ID; insert blood gas information; insert and update cardiac output; perform ALPE test; recognize and accept advice; use simulation and history interfaces; identify start of SBT process and status; and extubation assessment window. After completion of the BEACON Caresystem super user training questionnaire a super user training certificate was provided enabling the staff to cascade the training to the clinical teams in their unit. Similarly, after the user training a questionnaire was completed by the trainees and training recorded.

24/7 help hotline was setup and available to all site and supportive documents were provided to each site and when appropriated attached to the devices for quick reference:

BEACON Caresystem IFU- Operators Manual UK 1.0

BEACON Caresystem brochure UK 1.0

Quick Guide BEACON Patient V500 setup UK 1.1

Quick Guide BEACON Patient EvitaXL setup UK 1.1

Quick Guide BEACON Actions and Error Handling UK 1.0

Quick Guide Manual ALPE measurement UK 1.0

Quick Guide BEACON Blood Gas process and errors UK 1.0

Quick Guide BEACON patient Maquet setup UK 1.0

Quick Guide BEACON Tube setup and Maintenance UK 1.0

Electronic Case Report Form (eCRF) training (Smart-Trial)

Log in credentials and training in the eCRF was provided to the relevant study personnel with the right permissions; live demonstration was performed and SOPs – Using SMART-TRIAL in iCareWeanLean.v1 and iCareWean_Standard Operating Procedure ICU data for eCRF v1.0.

Troubleshooting strategies were disseminated with all sites through regular communication. Study and training updates were maintained through regular refreshing training sessions, education flyers, multisite meetings, newsletters and safety monitoring.

**S.8) Research governance**

The Research & Development (R&D) team at Chelsea & Westminster NHS Foundation Trust (CWFT) Sponsored the iCareWean Trial and Chair the Data Safety Monitoring Board (DSMB). The R&D team at the CWFT adhered to the NHS indemnity liability scheme in line with UK Good Clinical practice code of practice.

**Ethics & dissemination:** Approved by regional ethics committee of the HRA, REC reference: 17/LO/0887. IRAS reference: 226610.

**Trial Registration details:** ClinicalTrials.gov under NCT03249623. This research is registered with the National Institute for Health Research (NIHR) under CPMS ID: 34831.

**ESICM endorsement:** The ESICM Research Committee endorsed the project on 6^th^ July 2017.

**S.9) Data on cause of lack of eligibility**

The study design and projected time period was based upon extrapolation of the 2016 ICU admission data from two London-based NHS hospitals which provided a sample size of 548 patients over an 18-month period with a ventilation duration greater than 24 hours. Assuming that 50% of these patients were both eligible and consented, the study was designed such that 274 patient could be randomized to 137 in each the control and intervention groups, which was sufficient to power the study for detection of a 2 day, or 30%, reduction in ventilator time (Vizchaychipi [1]). It was expected that 710 patients would be screened to achieve this recruitment.

In practice, only 252 of the 985 patients screened were eligible for the study with a further 50% loss due to lack of consent. The causes of ineligibility which excluded more than 30 patients are provided in the following table.

Table S.9.1 – Reasons for exclusion.

| **Cause of exclusion*** | **Number** |
| --- | --- |
| Primary neurological patients | 288 |
| MV expected < 24hours | 259 |
| Haemodynamically unstable | 179 |
| Ventilation Mode not compatible | 72 |
| Multiple medical ICU admissions | 57 |
| End stage liver disease | 50 |

* Causes are not mutually exclusive.

The main reason for exclusion were for patients who’s primary disorder was neurological or patients who required mechanical ventilation for least than 24h following admission to the ICU. The definition of these two leading causes for exclusion from the study may have been ambiguous which led to clinical team interpretation of these criteria. Another plausibility is that indeed patient population admitted to the ICU has changed as identified during the screening process throughout the study.

**S.10) Evaluation of advice use.**

By recording the time periods and nature of advice provided by the Beacon Caresystem, along with the changes in ventilator settings, it is possible to evaluate how often the advice was followed. To do so requires defining the different types of advice and what it means to follow or not follow an advice. These definitions and the resulting evaluation of use of the system are provided here.

An advice was evaluated as “actionable” if it suggested changing ventilator settings from current conditions. The Beacon Caresystem often provides advice to remain at current conditions and these were not defined as “actionable”. In addition, the system can provide advice on a subset of ventilator settings, for example FIO_2_ alone, with other settings suggested to remain as is. In this case only the specific setting is considered actionable. The function of the BC means that the patient is evaluated every minute. Advice calculated to be the same from minute to minute which is only presented to the user on a single occasion is counted as a single advice.

For “actionable” advice the following possibilities are defined

*Followed*: An advice is considered followed if acted upon within a 2 hour window of being presented. Advice is considered followed if the setting is adjusted in the same direction as the advice. If the advice includes changing two settings, for example both tidal volume and respiratory rate, then the advice is only considered followed if both are changed in the correct direction within this duration.

*Not acted on*: An advice is considered not acted o n if the 2 hour window is exceeded without any ventilator change.

*Settings different than advised*: If ventilator settings are changed in a different direction to those advised within the 2 hour window. If advice includes changing two settings, and only one is changed within the time window this is classified as Settings different than advised. The logic of this decision is that if advice suggests decreasing tidal volume but increasing respiratory rate, then only decreasing tidal volume is not a similar clinical intervention. The same applies for a combination of FIO_2_ and PEEP advice.

*Remain*: Occasionally an advice to change settings is changed to an advice to remain at current settings during the 2hr window, thus becoming non-actionable. In this case the advice is classified as Remain and not evaluated as part of actionable advice.

*Cancelled*: If following advice a ventilator clinical event – extubation or mode change- occurs within the 2 hour window then the advice is considered no longer actionable and not evaluated as part of actionable advice.

Four types of advice are considered

Volume control – In volume control mode advice can be for tidal volume, respiratory frequency or a combination of these two settings.

Pressure control – In pressure control mode advice can be for pressure level, respiratory frequency or a combination of these two settings.

Pressure support – This is defined as advice on PS alone.

Oxygen – This is advice in any ventilator mode on FIO_2_, PEEP or a combination of these settings.

Figure S.10.1 illustrates a graphical output from the automated advice analysis process. This illustrates the latter portion of the pressure support reduction of the patient illustrated in figure S.5.1 inset B, of this ESM. Pressure support levels are given in red. System’s advice is shown as black crosses. Dashed verical lines illustrate the identification of a new actionable advice. This advice can be calculated and recorded several times, so the system identifies the last time it is shown as a dotted vertical line. Solid vertical lines indicate an action performed following an advice and are automatically annotated. The annotation “Followed” is illustrated here indicating that the advice falls into the followed category. Non-actionable advice are also marked as black crosses, and are annotated. The non-actionable advice “Advice is current” is annotated here, illustrating that the system agrees with current ventilator settings. Spreadsheets were automatically generated from these analysis counting the number of classifications of actionable and non-actionable advice.

Figure S.10.1 – A graphical example of automated advice analysis. Red line- pressure support values, black crosses – presentation of advice, annotated vertical lines – indication of response, including for this example advice followed, advice is current.

Table S.10.1 illustrates the results of the advice analysis. For each of the four types of advice, the percentage of actionable advice is given for advice “Followed”, “Not acted upon”, or where “Settings different from advised.”

Table S.10.1 – Analysis of the use of advice.

|  | Percent of total advice | Followed (% of type) | Not acted upon (% of type) | Settings different from advised (% of type) | Total (type) |
| --- | --- | --- | --- | --- | --- |
| Volume control | 7 | 76 | 21 | 3 | 100 |
| Pressure control | 6 | 76 | 17 | 7 | 100 |
| Pressure support | 26 | 93 | 6 | 1 | 100 |
| Oxygen | 61 | 89 | 8 | 3 | 100 |
| total | 100 | 88 | 9 | 3 | 100 |

Notes:

For volume and pressure control advice, all advice where settings were different from advice, was for advice on a combination of either Vt/PC and RR. For 88% of these one of the two advice settings was modified correctly, either not modified 50% or modified in the opposite direction 38%. For the remaining 12% one setting was modified in the opposite direction.

For PS, 75% of setting different from advice was due to a decrease following an advice to increase with the remainder an increase following advice to decrease. In half these cases, PS levels were returned to start values subsequent to the change.

For oxygenation, 73 % of settings different from advice was due to a reduction in FiO2 following advice to increase. 13% were due to a increase in FiO2 following advice to reduce. 10% was due to in increase in FiO2 following advice to increase both PEEP and FiO2, and 4% was due to a reduction of FiO2 following advice to reduce PEEP.

**S.11) Post-hoc analysis of high tidal volumes in control mode, and high SpO2 values.**

Figure 3, D and E illustrate substantial numbers of patients with high values of tidal volume in control mode ventilation. The majority of these patients were ventilated in pressure control mode and as such there is no set tidal volume to compare across groups. In addition, it is likely that many of these patients had substantial respiratory muscle effort, meaning that the high tidal volume may not be due to the pressure set on the ventilator but rather the pressure generated by respiratory muscles.

This post hoc analysis was performed to understand whether the elevated values of tidal volume might be due to respiratory muscle activity in control mode. If so, high tidal volumes would be seen at relatively low inspiratory pressures delivered by the ventilator. To assess this, the effective lung compliance was calculated, i.e. the ratio between measured tidal volume and delivered pressure by the ventilator. For patients without respiratory muscle activity values of compliance should be in the range typical of ICU patients, i.e. 20-50 ml/cmH2O. If respiratory muscles are active effective compliance will be above these levels. This does not reflect the state of the lung, but does provide an indication as to muscle activity.

Figure S.11.1 illustrates the effective compliance against tidal volume as averages over the period of control ventilation in each patient. Patients with tidal volumes > 10 ml/kg PBW had a tendency to elevated values of effective compliance, suggesting that high tidal volumes are more likely due to respiratory muscle activity than high values of inspiratory pressure set on the ventilator.

Figure S.11.1 – tidal volume versus effective compliance. Crosses indicate control group patients, circles intervention group patients.

In addition as seen on figure 3C of the main paper, a substantial proportion of patients SpO2 values were elevated. A post hoc analysis was performed to understand whether this was due to elevated FIO_2_ levels or relatively normal gas exchange in the lung.

Figure S.11,2 and S.11.3 plot the inspired oxygen fraction against the SpO_2_ level for control and support modes respectively. It can be seen in both groups that elevated SpO_2_ levels were typically at very low inspired oxygen fractions illustrating that these were due to normal gas exchange and not hyperoxaemic FIO_2_ levels. Points not lying around the diagonal from top left to bottom right indicate either high levels of FIO_2_ used to obtain high saturation levels, or low levels of FIO_2_ accepted despite low saturation levels, both of which can be considered sub-optimal.

Figure S.11.2 – Inspired oxygen fraction volume versus oxygen saturation for control mode ventilation. Crosses indicate control group patients, circles intervention group patients.

Figure S.11.3 – Inspired oxygen fraction volume versus oxygen saturation for support mode ventilation. Crosses indicate control group patients, circles intervention group patients.

**S.12) References**

1. Vizcaychipi MP, Martins L, White JR, et al. Intensive Care Weaning (iCareWean) protocol on weaning from mechanical ventilation: a single-blinded multicenter randomized control trial comparing an open-loop decision support system and routine care, in the general intensive care unit. BMJ Open. 2020 Sep 2;10(9):e042145.

2. Karbing DS, Spadaro S, Dey N, et al. An open-loop, physiologic model-based decision support system can provide appropriate ventilator settings. Crit Care Med 2018;46:e642–8.

3. Spadaro S, Karbing DS, Dalla Corte F, et al. An open-loop, physiological model based decision support system can reduce pressure support while acting to preserve respiratory muscle function. Journal of Critical Care, 2018, 48: 407–413

4. Rees SE, Karbing DS. Determining the appropriate model complexity for patient-specific advice on mechanical ventilation. Biomed Tech 2017;62:183–98.

5 Rees SE, Kjærgaard S, Thorgaard P, Malczynski J, Toft E, Andreassen S. The Automatic Lung Parameter Estimator (ALPE) system: Non-invasive estimation of pulmonary gas exchange parameters in 10-15 minutes. Journal of Clinical Monitoring and Computing, 2002, Vol 17, No.1, pp 43-52.

6. Thomsen LP, Karbing DS, Smith BW, Murley D, Weinreich UM, Kjærgaard S, Toft E, Thorgaard P, Andreassen S, Rees SE. J Clin Monit Comput. 2013, June 27(3), 341-50.

7. Karbing DS, Kjærgaard S, Andreassen S, Espersen K, Rees SE. Minimal model quantification of pulmonary gas exchange in intensive care patients. Medical Engineering and Physics, 2011, 33_240-248.

8. Rees SE, Allerød C, Murley D, Zhao Y, Smith BW, Kjærgaard S, Thorgaard P, Andreassen S. Using physiological models and decision theory for selecting appropriate ventilator settings. Journal of Clinical Monitoring and Computing, 2006;  Dec;20(6):421-429.

9. Rees SE, Spadaro S, Dalla Corte F, Dey N, Brohus JB, Scaramuzzo G, Lodahl D, Winding R, Volta CA, Karbing DS. Transparent decision support for mechanical ventilation using visualization of clinical preferences. Biomed Eng Online. 2022, in press

**S.13) List of investigators and acknowledgements**

List of investigators

Suveer Singh, Magill Department of Anaesthesia and Intensive Care Medicine, Chelsea & Westminster Hospital, APMIC, Surgery & Cancer, Imperial College London

Jaime Carungcong, Research and Development Delivery Team, Chelsea & Westminster Hospital.

Margaret Smyth

Patricia Costa, Research and Development Delivery Team, Chelsea & Westminster Hospital

Rhian Bull, Research and Development Delivery Team, Chelsea & Westminster Hospital

Kribashnie Nundlall, Research and Development Delivery Team, Chelsea & Westminster Hospital

Maria Flores, Research and Development Delivery Team, Chelsea & Westminster Hospital

Amrinder Sayan, Research and Development Delivery Team, West Middlesex University Hospital

Mini Thankachen, Research and Development Delivery Team, West Middlesex University Hospital

Marie-Louise Svesson, Research and Development Delivery Team, West Middlesex University Hospital

Dhanalakshmi Bakthavatsalam, Research and Development Delivery Team, North Middlesex University Hospital

Georgina Randell, Research and Development Delivery Team, Liverpool University Hospital NHS Foundation Trust

Karen Williams, Research and Development Delivery Team, Liverpool University Hospital NHS Foundation Trust

James White – location unknown.

Sponsor:

Damon Foster, Research and Development at Chelsea & Westminster NHS Foundation Trust

Rosalie Wagener, Research and Development at Chelsea & Westminster NHS Foundation Trust

Funder:

Mermaid Care A/S, Nøressundby, Denmark

Acknowledgements:

The ICareWean is eternally thankful to all members of the intensive care teams, patients and relatives from Chelsea & Westminster NHS Foundation Trust, West Middlesex University Hospital, North Middlesex University Hospital, Norwich and Norfolk University Hospital Foundation Trust and Liverpool University Hospital NHS Foundation Trust. A special thank you to Sandhiya Mandalia, Statistical Scientist from IQVIA Connected Intelligence. We would also like to acknowledge the technical expertise and support of Søren Thomsen and Jakob Brohus from Mermaid Care A/S.
